# Effects of Mecp2 loss of function in embryonic cortical neurons: a bioinformatics strategy to sort out non-neuronal cells variability from transcriptome profiling

**DOI:** 10.1186/s12859-015-0859-7

**Published:** 2016-01-20

**Authors:** Marcella Vacca, Kumar Parijat Tripathi, Luisa Speranza, Riccardo Aiese Cigliano, Francesco Scalabrì, Federico Marracino, Michele Madonna, Walter Sanseverino, Carla Perrone-Capano, Mario Rosario Guarracino, Maurizio D’Esposito

**Affiliations:** 1 Institute of Genetics and Biophysics “A. Buzzati Traverso”, National Research Council (CNR)-80131, Naples, Italy; 2 Laboratory for Genomics, Transcriptomics and Proteomics (LAB-GTP), High Performance Computing and Networking Institute (ICAR), National Research Council (CNR)-80131, Naples, Italy; 3 Sequentia Biotech SL, Calle Comte D’Urgell, 240 08036 Barcelona, Spain; 4 IRCCS Neuromed, via dell’Elettronica, Pozzilli (Is), Italy; 5 Department of Pharmacy, University of Naples Federico II, Naples, Italy

**Keywords:** Rett syndrome, Embryonic cortical neurons, Primary branching, Neural cells, MeCP2, RNA-sequencing

## Abstract

**Background:**

Mecp2 null mice model Rett syndrome (RTT) a human neurological disorder affecting females after apparent normal pre- and peri-natal developmental periods. Neuroanatomical studies in cerebral cortex of RTT mouse models revealed delayed maturation of neuronal morphology and autonomous as well as non-cell autonomous reduction in dendritic complexity of postnatal cortical neurons. However, both morphometric parameters and high-resolution expression profile of cortical neurons at embryonic developmental stage have not yet been studied. Here we address these topics by using embryonic neuronal primary cultures from Mecp2 loss of function mouse model.

**Results:**

We show that embryonic primary cortical neurons of Mecp2 null mice display reduced neurite complexity possibly reflecting transcriptional changes. We used RNA-sequencing coupled with a bioinformatics comparative approach to identify and remove the contribution of variable and hard to quantify non-neuronal brain cells present in our in vitro cell cultures.

**Conclusions:**

Our results support the need to investigate both Mecp2 morphological as well as molecular effect in neurons since prenatal developmental stage, long time before onset of Rett symptoms.

**Electronic supplementary material:**

The online version of this article (doi:10.1186/s12859-015-0859-7) contains supplementary material, which is available to authorized users.

## Background

*Mecp2* encodes a methylated DNA-binding protein and is the causative gene of Rett syndrome [1] (RTT, MIM #312750) a progressive neurodevelopmental disorder affecting 1:10,000 females worldwide [2]. Patients are apparently healthy during the first 6–18 months of life, then the disorder causes mental retardation, deceleration of head growth, seizures, motor dysfunction, hand stereotypies together with many other disabling symptoms [3]. *Mecp2* is ubiquitously expressed with 2 splicing isoforms, Mecp*2*A and B [4, 5]. In the brain these isoforms are developmentally and regionally regulated, with Mecp*2*B (also known as Mecp2α or Mecp2_e1) more abundant in adulthood. Both in humans and mice the delayed onset of RTT symptoms is in accordance with the postnatal increase of the Mecp2B protein [6].

Neuronal morphology plays a significant role in determining how neurons function and communicate. Specifically, it affects the ability of neurons to receive inputs from other cells and contributes to propagation of action potentials. The morphology of neurites also affects on how information is processed by the brain [7, 8]. Like many disorders associated with mental retardation [9], neuropathology of RTT reveals an increase in neural density due to decrease in cell size, together with reduced neurite length and branching in post mortem brains, as well as in *Mecp2*-loss of function mutant mice (*Mecp2*-null). Delayed development of neuronal morphological features and maintenance of functional networks [10] in *Mecp2*-null mice suggest that MeCP2 impacts neuronal maturation [11].

More recent studies have shown that RTT is not exclusively a neuronal disease. Despite the low level of MeCP2 expression in astroglia, oligodendroglia and microglia [12, 13], conditionally *Mecp2* loss in brain non-neuronal cells detrimentally influences dendritic integrity, synapses and protein expression in neurons [12, 14, 15].

Availability of many RTT mouse models [16] contributed to disentangle MeCP2 functions and demonstrate that many RTT symptoms can be reverted by postnatal re-activation of MeCP2 expression [17].

Initially described as a transcriptional silencer through the binding to methylated promoters and histone deacethylases recruitment, extensive studies in mouse models have depicted MeCP2 as a multifunctional protein able to activate gene expression, model nuclear architecture, and regulate alternative splicing and translation [18, 19]. In particular, the precise role of MeCP2 as a transcription modulator is still under investigation. Expression profiling of whole brain homogenates from *Mecp2* null mice and postmortem brains from RTT patients, revealed only subtle differences. Most productive efforts to identify MeCP2 regulated-targets took into account the problem of brain heterogeneity, in terms of regionality and cellularity, as a dilution factor which leads to underestimate the actual number of deregulated genes [20].

Studies conducted till this date, only evaluate post-natal transcriptional profiling as well as neuronal morphological parameters in RTT mouse models. Current hypothesis claims that key embryonic and perinatal developmental steps are not altered until presymptomatic stage (3–5 weeks in *Mecp2* null male mice). A candidate gene approach study has shown that expression of important proteins for neuronal maturation (i.e. ID1 and ID2, inhibitor of differentiation) is impaired at embryonic day 15 (E15) in the cerebral cortex of *Mecp2* null male mice, transiently (ID2) or with a long-lasting effect (ID1) over postnatal stages [21]. Furthermore, MeCP2 has been shown to promote heterochromatin reorganization during neural differentiation of embryonic stem cells and in mouse primary cortical neurons [22, 23]. In turn, chromatin organization changes are essential for neuronal development, as they impact on gene expression.

In light of these findings, in this study we investigate the effects of *Mecp2* loss of functions in earlier developmental stages than those analysed so far. By performing morphological analyses of primary embryonic cortical neurons dissected from *Mecp2* null mouse brains we show reduced neuritic arborization due to *Mecp2* loss of function. We profiled embryonic wild type (WT) and *Mecp2*-/Y (null) gene expression using RNA-sequencing assays. The availability of cell-type specific datasets, allowed us to use bioinformatics methods to isolate neuron enriched deregulated genes affected by MeCP2 deficiency. Results from our study suggest a role of non-neuronal brain cells variability for reproducibility and validity of gene expression profiling readouts.

## Results

### Mecp2 deficiency impairs primary branching of embryonic cortical neurons

We examined primary cultures of neurons dissected from E15 cerebral cortices of *Mecp2* null embryos (*Mecp2*^tm1.1Bird/J^ strain) to investigate whether changes in neuronal morphology already occur during early brain development of RTT mouse models. Since *Mecp2* starts to be expressed in cortical layers at E14.5 [24] mostly in early neuronal committed cells [6], we chose this stage to dissect cortices of *Mecp2* null and WT littermates. We considered embryonic neuronal primary cultures a useful tool to address our topic, as considerable information has been gathered on their neuronal development and maturation [25].

**Fig. 1:**
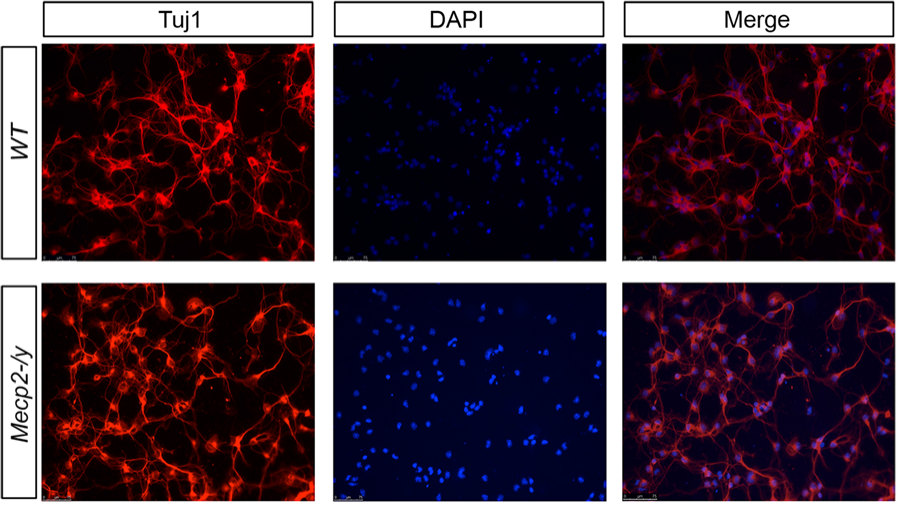
Immunocytochemical staining of cortical primary cultures derived from WT and *Mecp2* null mouse E15 embryos. Cortical neurons were immunostained using an anti- Tuj1 antibody (red) and representative images are shown. Cell bodies were counterstained with the nuclear marker DAPI (blue). Magnification: 20x; Leica DMI6000 B inverted microscope

Primary cultures are often heterogeneous and require characterization of their cell types (neurons versus non-neuronal cells, i.e. glia) and neuronal sub-types. These latter may be identified through neurotransmitter synthesized- and/or region-specific markers. The phenotype of our cells was investigated by immunofluorescence experiments performed with cell-type specific antibodies. Cells were labeled with anti- β-tubulin-III (Tuj1, neuronal marker), GABAergic neurons with anti-GABA (γ- aminobutyric acid) and astrocytes with anti-GFAP (glial fibrillary acidic protein). More than 90 % of cortical cells in WT and *Mecp2* null mouse cultures were neurons, as judged by counting the Tuj1 positive cells counterstained with the nuclear marker DAPI (Fig. 1). The percentage of GABAergic positive neurons over Tuj1 positive cell was about 30–35 %, whereas GFAP-positive cells were rare (about 1 % of DAPI stained nuclei). An additional figure file shows this in more detail [see Additional file 1].

**Fig. 2:**
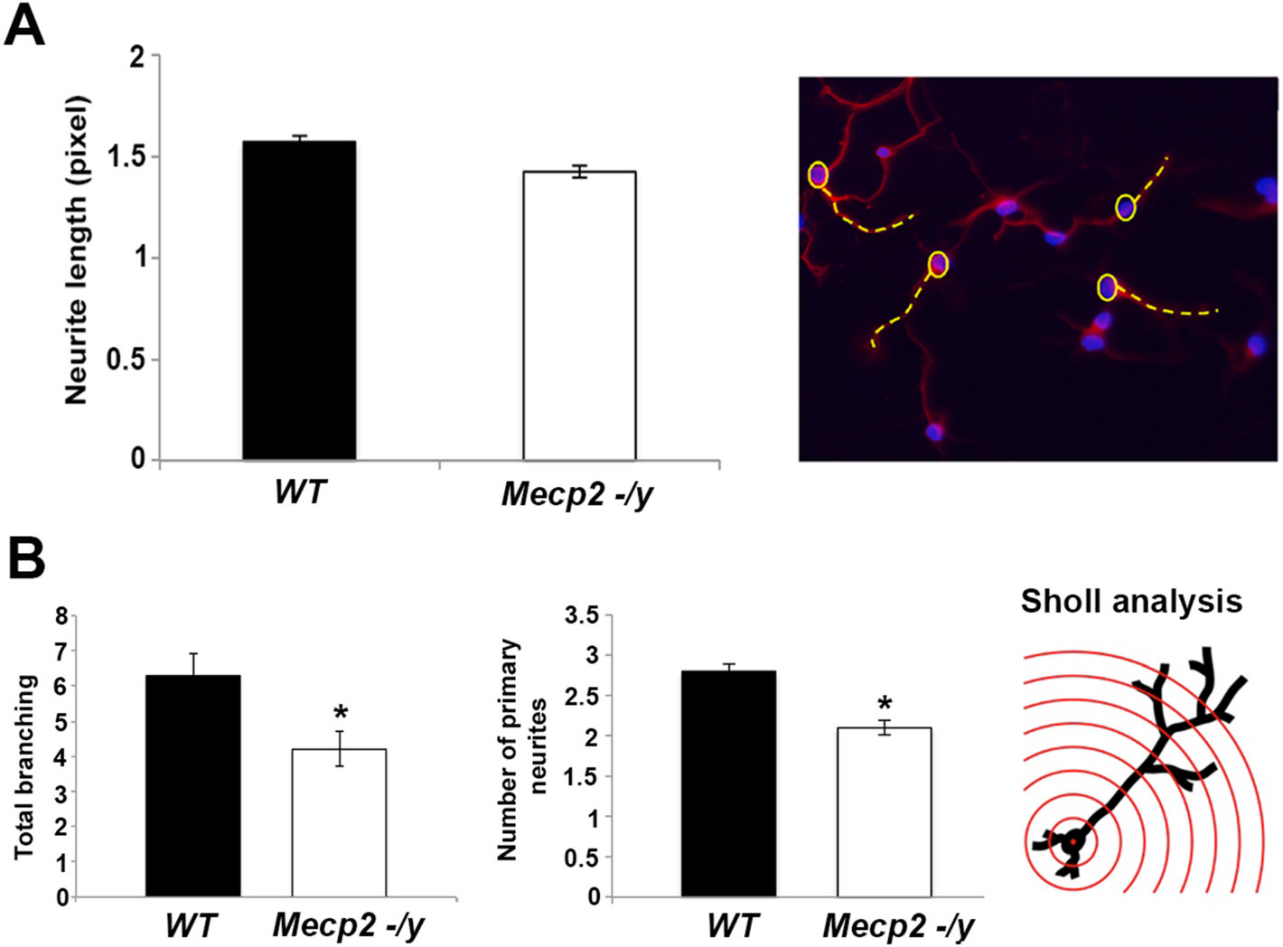
Morphometrical analysis of cortical primary cultures from WT and *Mecp2* null mouse E15 embryos. Neuronal primary cultures were used to compare morphological parameters between WT and *Mecp2* null condition. **a** Neurite length was measured on cells stained with anti-Tuj1 antibody (ImageJ software). The image on the right shows a representative Tuj1 immunostaining (red) of cortical neurons counterstained with the nuclear marker DAPI (blue). The operator manually drew the dashed yellow line from the soma (yellow circle) to the end of the primary neurite in order to measure neurite length. **b** Total and primary branching was evaluated by Sholl analysis. Its internal algorithm creates a series of consecutive concentric circles centered on the soma of the neuron and counts the number of neurites crossing these circles (drawn on the right side). The bars represent means ± SEM from randomly selected fields for each cell culture condition (*n* = 8). * *p* < 0.05

Subsequently to characterization, cortical cells were stained with Tuj1 antibody and morphometrically analyzed. We will refer to *neurites* because cells were in vitro cultured for only three days, not enough time to distinguish axons from dendrites. The length of the neurites was estimated by measuring the distance from the cell soma to the end of the primary neurites (neurites that originate directly from soma). Neurite branching was estimated by Sholl analysis, one of the most commonly used methods to quantify neuronal dendritic complexity [26]. We find that the length of neurites does not differ between WT and *Mecp2* null neuronal cultures (Fig. 2a). On the contrary, total branching and primary branching (i.e. the number of secondary neurite portion/fragment growing from the main neurite extension) are significantly lower in *Mecp2* null neurons compared to age-matched WT control (Fig. 2b).

### Mecp2 deficiency impairs gene expression of embryonic cortical cells

Primary cell cultures from WT and *Mecp2* null mice were profiled by RNA-sequencing to obtain a high- resolution dataset and search for cortical genes possibly affected by MeCP2 loss during early mouse development.

As *Mecp2* is an X-linked gene, we collected only male embryos to avoid the confounding effect of X chromosome inactivation typical of females on gene expression profiles. The inbred C57Bl/6 J strain was used to minimize genetic background influence. In order to minimize inter-individual variability, RNA isolated from cells of single embryos was pooled accordingly to *Mecp2*-genotypes in three samples/genotype (see Methods) and RNA from each sample was sequenced.

We obtained 30 million of reads per sample with quality scores higher than Q20, mapping on average 96 % of them to Mus musculus reference genome sequence (GRCm38). A hierarchical clusterization based on correlation was performed to check the quality of the samples (an additional figure file shows this in more detail [see Additional file 2]). One null and one WT samples did not cluster properly (data not shown), thus they were excluded from further analysis.

Total reads related to *Mecp2* were comparable among WT samples (RPKM values: 10.21 and 9.22 for WT2 and WT3 respectively) suggesting similar levels of expression. In null samples, few *Mecp2* specific reads (RPKM values: 0.90 and 0.84 for N2 and N3, respectively) mapped exclusively downstream to the deleted part of the gene. In addition, we found intergenic reads spanning the 3 kb genomic region from *Mecp2* 3′UTR to *Irak1* locus (an additional figure file shows this in more detail [see Additional file 3, left panel]). The counts per million (CPM) of the reads mapping in this region was 5.5 and 0.04 for *Mecp2*-/Y and WT samples, respectively. The observed difference was found to be statistically significant after Welch Two Sample t-test (*p* <0.05) and transcription of this region was confirmed by RT-PCR with two different sets of primers ([see Additional file 3, right panel]).

**Fig. 3:**
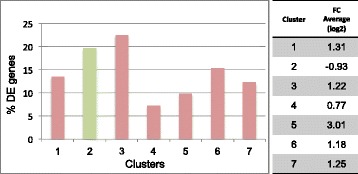
Percentage distribution of differentially expressed (DE) genes across clusters of FC average. K-Means clustering applying Pearson correlation as distance metric identifies 7 clusters based on the mean fold change of the 490 DE genes. Cluster_2 includes all down-regulated genes (green). Up-regulated genes (red) are enriched in Cluster_3 and broadly distributed in Cluster_1 and Clusters_ 4–7

As for the overall transcripts data set, by quantitative comparison and using a q-value (FDR) ≤0.05 we find 490 differentially expressed (DE) genes in E15 cortical neurons of null mice *versus* WT littermates, with 394 up-regulated (80 %) and 96 down-regulated (20 %) genes. DE genes cluster in 7 classes (Fig. 3) according to their expression profiles and their average fold-change (FC). Down-regulated genes show a mean FC of −0.93, whereas most part of up-regulated genes shows a mean FC of 1.22. Among up- regulated genes belonging to Cluster_3, we find *GFAP* (ENSMUSG00000020932) commonly referred to as an astrocyte marker. Even if GFAP has been already reported as a *Mecp2*-target in RTT patients [27] and in *Mecp2*-knock down rat model [28] this finding was not fully expected. Indeed *GFAP* was not found deregulated in primary astrocyte cultures prepared from postnatal day 1 cerebral cortex of the same null mouse strain [29] we used in this study. In fact, transient upregulation of *GFAP* in *Mecp2*-deficient cells seems to be female specific at least in amygdala and hypothalamus [28], whereas it increases in the hypothalamus of a male transgenic mouse overexpressing MeCP2 under its endogenous promoter [30].

*Aldh1L1* (ENSMUSG00000030088), another gene mapping to the same FC based cluster of GFAP (cluster_3), is up- regulated as well in null samples *versus* controls. *Aldh1L1* strongly labels many more astrocytes than *GFAP* [31]. This finding, together with the fact that primary cells were 90 % composed of neurons (see previous results above), suggested us to verify whether the expression of other non-neuronal markers occurred across profiled samples. Microglia and oligodendrocyte markers (*CD11B*-ENSMUSG00000030786 and *NG2/CC1*- ENSMUSG00000015478/ENSMUSG00000005871, respectively) are indeed expressed in our in vitro cellular system, even if their expression ranged from very low to slightly low levels (not shown).

### Bioinformatics cell sorting reveals neural differentially expressed genes at embryonic developmental stage

**Fig. 4:**
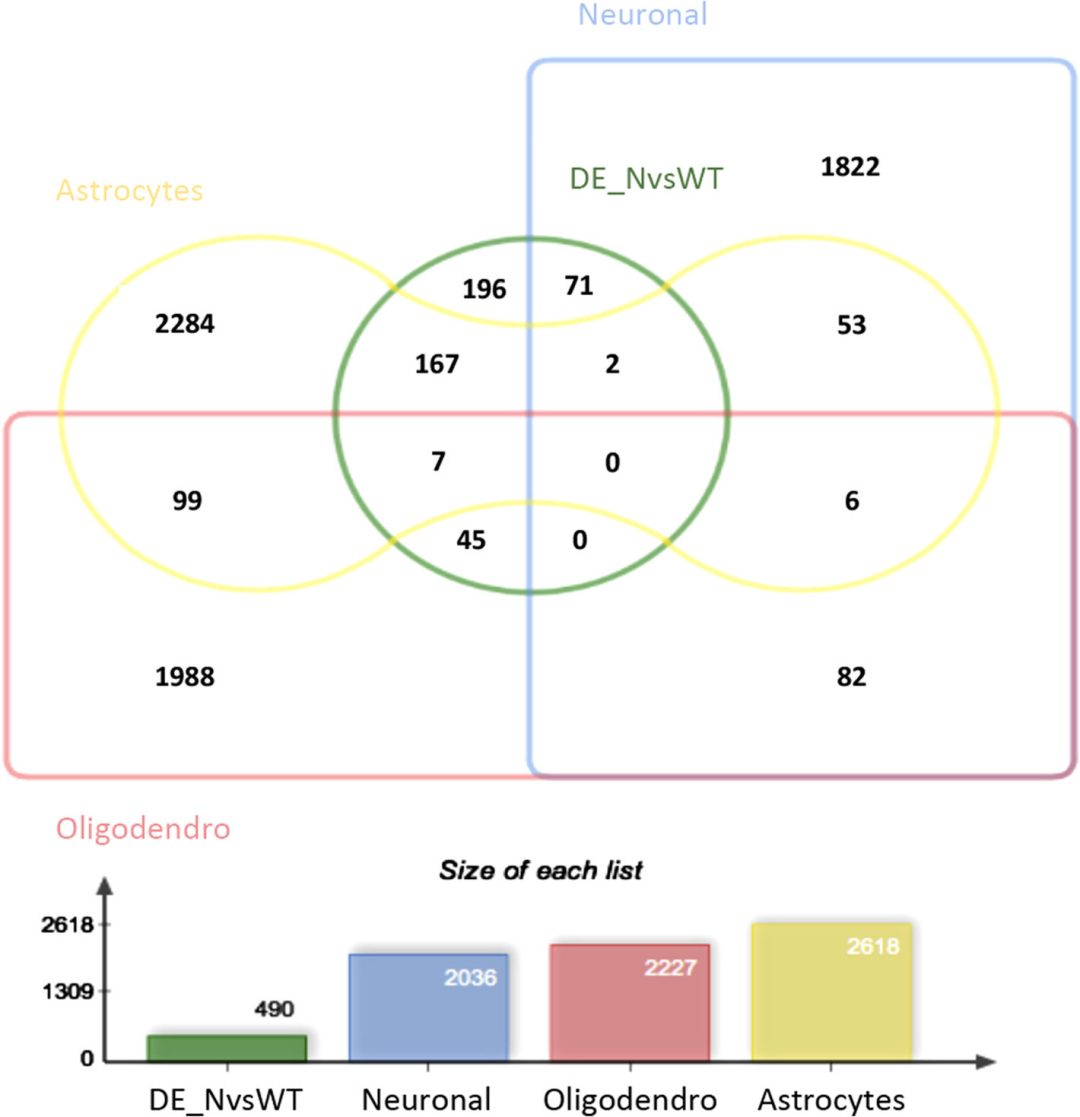
Distribution of the 490 differentially expressed genes in neural cells. The Jvenn diagram compares the 490 DE genes of this study (DE_NvsWT) with genes representing the transcriptional signature of neurons, oligodendrocytes and astrocytes (obtained from [31]). Focusing on genes specifically enriched in each cell-type we find: 71 neuronal genes, 167 astrocytes genes, 45 oligodendrocytes genes. Unmapped genes are 196, whereas 9 genes are shared between two cell- types

We next wanted to verify the hypothesis that some of the differentially expressed genes that we identified could be of non-neuronal origin. To do this we bioinformatically sorted the cells using available gene expression microarray data from the main central nervous system (CNS) neural cell types [31]. We map the 490 differentially expressed genes resulted from RNA- sequencing across three different neural cell types. Figure 4 shows that 34 % of DE genes found in this study may be collected specifically under the astrocyte-signature (167/490), 9 % (45/490) under oligodendrocyte- cell type, while 14 % (71/490) of genes may be enriched in neuronal cells. We classify the remaining 196 DE genes (40 %) as unmapped. Most of these latter genes (168/196, see Additional file 4) were present on the GeneChip microarray (GSE9566) used in Cahoy’s study [31] but probably they have not any cell type specificity. Moreover, as the expression of microglia marker-*CD11B/Itgam* was found among the 196 unmapped genes, we compared them to the recently reported microglia sensome [32], without finding yet any overlapping gene.

#### Differentially expressed genes with astrocyte transcriptional signature

Firstly, we compared the list of the 167 genes enriched in astrocytes with those specifically deregulated in postnatal primary cultures of *Mecp2*-/Y astrocytes [29]. Only 3 genes overlap with our astrocyte-enriched dataset: *Gabrg1* (ENSMUSG00000001260), encoding GABA-A receptor gamma 1 concordantly deregulated in both datasets; *Aldoc* (ENSMUSG00000017390) and *Fgfbp3* (ENSMUSG00000047632) that are discordant with results of Yasui ([see Additional file 5]). This finding, together with the fact that two astrocyte markers such as *GFAP* and *Aldh1L1* (see above) have been found both up-regulated in our *Mecp2*-null derived primary cultures *versus* controls, raises the possibility that a larger amount of GFAP+ and Aldh1L1+ cells (even if rare) were originally present in the null compared to the WT cultures from which RNA for sequencing was isolated.

#### Differentially expressed genes with oligodendrocyte transcriptional signature

Among the 490 DE genes only 9 % are enriched in oligodendrocytes (Fig. 4). We find comparable expression of *NG2*, a marker of oligodendrocyte precursor cells, and *CC1*, a marker of mature oligodendrocytes. These markers are not differentially expressed with the respect to the genotype of samples (see Additional file 5).

According to Gene Ontology (GO) analysis (An additional composite figure file shows this in more detail [see Additional file 6A]) the most significantly represented biological processes are those related to ensheathment of neurons (especially myelination) and regulation of action potential (GO: 0042552 and 0019228, respectively with *p*-value ≤ 0.05). Given that the transcriptome dataset [31] profiles cells in postnatal mouse, GO outcomes would have been biased towards biological process more active in postnatal stages, as myelination does.

Nonetheless, GO together with Panther analysis (see Additional file 6B) give us interesting directions of investigation to be tested, as the most enriched annotations in this category of genes are related to protein families involved in oligodendrocytes differentiation, such as Olig 1 and 2 [33], CD82 [34] and CD9 [35], or to lipid synthesis ([see Additional file 6A]), such as ELOVL7 [36] whose expression increases in null cells here sequenced ([see Additional file 5]). To be noticed, aberrant oligodendrocytes maturation could affect neuronal-non neuronal cells interactions [15] as reported for astroglia. Furthermore, among the 45 deregulated genes within oligodendrocyte- cell type no matches have been found in the cerebral cortex dataset of developmentally regulated genes [37] suggesting that their expression level might be constant during brain development.

#### Differentially expressed genes with unrecognized transcriptional signature

Functional annotation of the 196 DE genes not mapped to neurons or glial cells shows that they are significantly enriched in biological process like the Tumor necrosis factor (TNF)-mediated signaling pathway (An additional figure file shows this in more detail [see Additional file 7). In response to numerous stimuli, TNF-pathway triggers downstream effects related to cell viability, gene transcription, ion homeostasis and synaptic plasticity. This pathway as well as activated microglia are key mediators of neuroinflammation and may contribute to neuronal dysfunction.

This functional outcome may have important molecular implications on the neuronal morphological phenotype here described, as it has been shown that postnatal *Mecp2* null mouse microglia detrimentally affects dendrites and synapses by releasing an excess of glutamate [14].

In addition, as perturbation of embryonic microglial activity impairs normal assembling of cortical networks [38] there could be a causal relationship between MeCP2-deficient microglia and forebrain connectivity even during embryonic development.

The microglial marker *CD11B* indeed maps to this category but it results to be up-regulated in our *Mecp2* null cell cultures versus WT control, possibly indicating a different distribution of *CD11B*+ cells across profiled samples, as another report indicates that *CD11B* is not altered by *Mecp2* deficiency [14].

#### Differentially expressed genes with neuronal transcriptional signature

Of the 71 genes assigned to neuronal cells, forty-three genes (60 %) are more expressed in *Mecp2* null cells than in WT while the remaining twenty-seven genes (40 %) are down-regulated ([see Additional file 5]).

Before further characterizing these genes we checked whether neuronal and GABAergic markers were equally expressed across samples. The overall number of neurons does not differ between WT and null samples, as previously shown by Tuj1-immunofluorescence staining (see above) and then confirmed at transcriptional level by RNA-sequencing (β-tubulin-III- ENSMUSG00000072235 RPKM values in WT samples are 1202.42, and 1081.06, respectively; in null samples 1043.82 and 1114.27, respectively). Similarly, there are no differences in expression level of: a) GABAergic marker-transcripts, such as GABA synthesis enzyme GAD2 (ENSMUSG00000026787, RPKM values in WT samples are 8.30 and 6.46, respectively; in null samples 6.51 and 6.58, respectively); b) GAD1 (ENSMUSG00000070880); c) genes encoding the GABA transporters, such as *Slc32a1* and *Slc6a1* together with Dlx1 and Arx [39] ([see Additional file 5]).

**Fig. 5:**
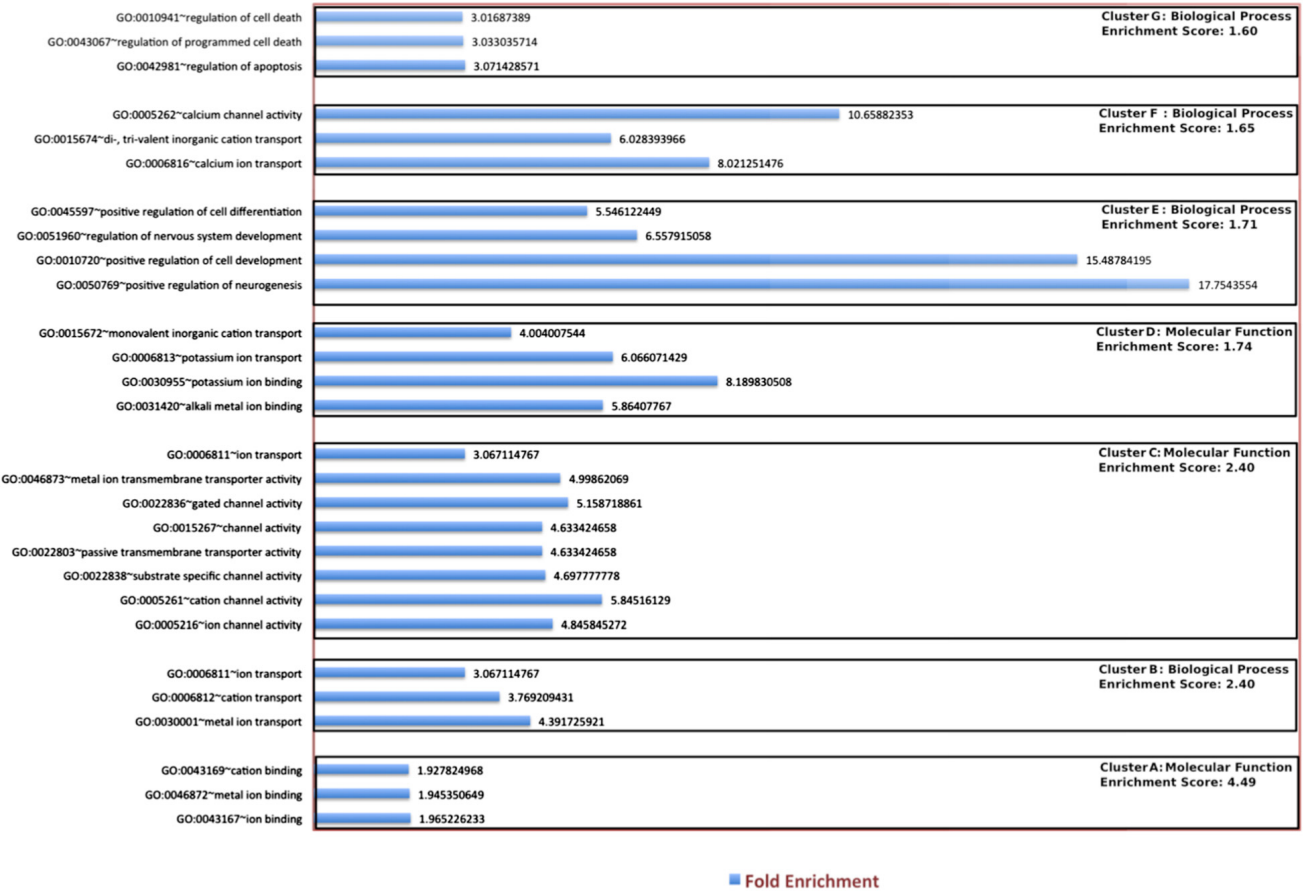
Functional annotation clustering of neuronal specific differentially expressed genes. Using Functional annotation clustering algorithm in-built in DAVID web resource, seven functional annotation clusters based on GO terms (biological process, molecular function and cellular components) were obtained within neuronal specific differentially expressed genes. Each cluster is significantly enriched with enrichment score cut-off value >1. Within each cluster, all the related gene ontological terms are also separately enriched for the given neuronal specific gene with Ease score value (see Methods) cut-off ≤ 0.05. Note that the most enriched biological process GO terms fall within clusters E and F and are related to specific neuronal functions. No cellular compartment cluster was sufficiently enriched

Assuming homogeneity of samples, the list of DE genes carrying a neuronal transcriptional signature was then analyzed, to search for functional categories more enriched and potentially targeted by *Mecp2* loss. Functional clustering (Fig. 5) shows 7 significantly represented clusters (*p*-value < 0.05), with three molecular function terms (clusters A, C and D) and four biological process annotations (clusters B, E-G). Interestingly, the major functional themes assigned are specifically related to neuron specific features, such as neurogenesis and cell differentiation (cluster E) together with neuronal activities. Indeed, ion binding (cluster A), transport (cluster B) and channel activity (Cluster C, D and F) altogether sustain synaptic transmission. Among genes listed in these functional clusters, some contribute to shape neuronal morphology, such as *Mef2C* and *Tiam1* (also identified in [30]). Another interesting gene found in these clusters is *GDA* encoding guanine deaminase, a protein involved in microtubule assembling [40] and regulation of dendrites number [41]. To further validate the GO and functional annotation results, we carried out enrichments analysis of each GO and functional terms separately for the given list of neuronal specific genes. We obtained enriched GO and functional terms along with multiple test corrected *p*-values (An additional table file shows this in more detail [see Additional file 8]). Together with DAVID annotation, we carried out GO annotation analysis through AmiGO 2, obtaining significant GO terms using Bonferroni correction method (*p*-value < 0.05). The results (see Additional file 8) confirm the biological inference for the neuronal specific genes obtained by DAVID analysis.

To understand whether the neuronal genes mis-regulation we found at embryonic stage is conserved throughout development, we compared our data with a number of gene expression studies profiling various brain regions of postnatal *Mecp2* mutant brain. To avoid any mouse background confounding effects, we focused our interest in regions dissected from the same *Mecp2* null mouse strain [30, 42] analyzed in this study. We find that the highest rate of sharing is achieved with hypothalamic genes: twenty-seven out of seventy genes (excluding *Mecp2*) are differentially expressed in postnatal hypothalamus of *Mecp2* null mice [30] with 12 genes (44 %) deregulated in the same direction ([see Additional file 5]). Only two genes are concordantly shared with cerebellum of *Mecp2* null mice, with *Pls3* (ENSMUSG00000016382) gene also present in the hypothalamus list ([see Additional file 5]). The pairwise comparison of the 71 neuronal DE genes with those published by Zhao and colleagues [43] in symptomatic P60 (postnatal day 60) *Mecp2* null mice (Bird strain) revealed only 4 genes in common (see Additional file 5). Nonetheless, all neuron related GO terms above mentioned are also shared between the two datasets, uncovering specific functions of MeCP2 regulated genes during development.

Screening the cerebral cortex dataset of healthy mice [37] we find 30 genes ([see Additional file 5]) merging with our neuronal enriched dataset. Notably, 90 % of them become more highly expressed in adult cortex during mouse development, including 7/8 deregulated genes concordant with hypothalamic findings.

Finally, we find that 50 % of neuronal enriched up-regulated genes (22/44, data not shown) are long genes (around or more than 100 kb) as already reported for MeCP2-repressed genes [20, 44].

## Discussion

Neuroanatomical studies in cerebral cortex of *Mecp2* null mice showed delayed maturation of neuronal morphology and autonomous as well as non-cell autonomous reduction of dendritic complexity of postnatal cortical neurons. In addition, abnormal cell body size and density of both glutamatergic [45–47] and GABAergic neurons [48] has been reported for a RTT mouse model [48] as well as for inhibitory neurons differentiated from human embryonic stem cells lacking *Mecp2* [49].

In this study, we describe effects of *Mecp2* loss of function both on the morphology and transcription profile of the embryonic neocortical neurons. Interestingly, we find a reduced neurite branching in *Mecp2* null neurons, whereas neurites length is not affected. Similar results have been recently described in inhibitory neurons differentiated from human embryonic stem cells lacking *Mecp2* [49]. In addition, we identify a neuronal subset of embryonic deregulated genes that are highly expressed in mouse adult cortex, possibly indicating that some subtle embryonic defects will be functionally amplified later in postnatal life when clinical RTT symptoms become evident. The findings in our study further support the hypothesis that *Mecp2* has a role in structural plasticity of cortical neurons and highlight the effects on the transcription of a subset of neuronal genes. It has been recently reported that *Mecp2* deficient mice display regional- and age-dependent variations in the GABA pathway and, to a lesser extent, in the glutamate pathway [50]. Focusing on functional annotations significantly enriched in neuronal cells here profiled, we achieved additional as well as interesting results: MeCP2 deficiency should impair the same biological process even across different brain areas and developmental stages, as suggested by the overlapping of Gene Ontology terms with those described for striatal deregulated genes of postnatal *Mecp2* null mice [43].

However, it would be interesting to measure in our cellular system the impact of non-neuronal cells on the transcription of neuronal genes. Indeed, the deregulated genes due to the *Mecp2* loss only in the striatal GABAergic neurons partially overlap with those impaired by a constitutive *Mecp2* loss [43].

Our bioinformatics approach on the list of differentially expressed genes in primary cultures from *Mecp2* null and WT cortices has another important outcome: on one hand allows to focus on genes with a neuron transcriptional signature, while on the other identifies non-neuronal enriched genes contribution to the list. Overall findings from non-neuronal cortical cells may suffer from inter- individual variability and chance to survive in serum-free culturing medium. Prior RNA-sequencing, pooling of samples (merely based on genotype and gender of embryos) may have masked such variability, giving rise only to partial conclusions of *Mecp2* loss of function effects. The simultaneous presence in our cultures of different neural populations allowed us to preserve cell-cell interactions between neurons and non-neuronal cells. This is a fundamental aspect in molecular and morphological studies of *Mecp2* null cells, as neuron-glia and -microglia interactions have emerged as a crucial point to control RTT progression and for some of them to reverse symptoms.

There is an extensive reorganization of mouse transcriptome between embryonic and postnatal developmental stages [37, 51]. Moreover, gene expression programs are highly divergent across cell types [52]. Likely, not overlapping results of downstream effectors of *Mecp2* deficiency can be also affected by the specific developmental window of profiling. In addition, it has been shown that sorting methods to profile specific cell types can themselves produce in some cases not comparable results [53]. Despite this, a small subset of deregulated neuronal genes here identified has already been described in a previous study [30] at a postnatal stage and in a different brain region (hypothalamus). We speculate that *Mecp2* loss may impact transcripts abundance of those genes in a space-temporal independent ways, starting from mid-gestation period. Interestingly, at E15 when those genes are deregulated in the cortex, MeCP2 protein is not detectable in hypothalamus [24].

Most part of neuron-enriched deregulated genes here found are up-regulated, whereas in previous studies most part of *Mecp2* deregulated genes are repressed. This discrepancy may be likely linked to different developmental stages analysed but also to the technology utilized, given that we were able to measure even very low expressed genes (see Fig. 3). However the rate of up-regulated genes here found is consistent with that reported by Sugino [20] based on cell type specific approach. Moreover, concordantly to Sugino et al. [20] and Gabel and et al. [44] we also find that genes up-regulated by Mecp2 loss are biased toward long genes.

In addition, RNA-sequencing permitted us to confirm that *Irak1* deregulation is causally linked to genetic structural manipulation of mouse *Mecp2* locus, rather than to functional loss of *Mecp2*. Indeed *Irak1* levels specifically increase in the Bird strain of *Mecp2* null mice lacking exons 3 and 4 [54], whereas in the Jaenisch strain is normally expressed [55]. In all brain regions to date profiled [30, 43, 55–57] and in this study, *Irak1* is always up-regulated. Our mapping of reads in the intergenic region between *Mecp2* and *Irak1* in null samples may be due to spurious transcription occurring in the absence of terminal coding part of *Mecp2* gene. It is thus reasonable to suppose that at least a subset of differentially regulated genes found in this study and by others should be considered as *Irak1*- dependent downstream effects rather than *Mecp2* targets. Among downstream effectors of *Irak1* signalling pathway there is *NF-Kb*, a transcription factor even acting as a modulator of neurite outgrowth [58]. Moreover, the virtual cell sorting we performed assigned *Irak1* to cells resembling activated microglia, characterized by TNF-mediated signal pathway enrichment. This finding supports an already proposed hypothesis of a constitutive activity of signalling pathways mediating the TNF- action, as *Mecp2* null microglia releases significantly less TNF-alpha under specific stimulation [14]. Remarkably, our findings motivate further studies focused on genes involved in shaping cell morphology, whose mis-expression can lead to altered formation, pruning and maintenance of neurites and synapses. *Mef2c* and *Tiam1*, already described as *Mecp2* target genes, may be good candidates. Mef2c negatively regulates synapses number and function [59] and is up-regulated by *Mecp2* loss (this study and [30]. Tiam1, essential for Rac1 activation and neurite outgrowth of cortical neurons [60], is slightly down-regulated in the cellular system we analyzed. As Tiam1 manifests the opposite response to *Mecp2* loss in postnatal hypothalamus [30], we can suppose that *Mecp2* may exert regional and/or stage specific regulation on specific genes. On the contrary, the up-regulation of the *GDA* gene may be in accordance with a model of compensatory strategy of neuron to recover branching defects [10], whereas the up-regulation of thyrotropin releasing hormone (TRH)-degrading enzyme would have a direct impact on action potential shaping of cortical neurons.

## Conclusions

To our knowledge dendritic complexity of embryonic cortical neurons, as well as the embryonic high- resolution expression profile was never examined in *Mecp2* null mice. Using a very sensitive technology we were able to profile even genes transcribed at low level, showing that our close to pure primary neuronal cultures derived from embryonic cortices may in fact contain low and poorly detectable amount of non neuronal cells. The virtual *a posteriori* cell sorting here described allowed also to focus specifically on differentially expressed genes of neuronal origin. We are confident that the cellular system here used could be further investigated, providing a fine characterization of neuronal subpopulation together with non-neuronal cells.

We thus suggest that abnormal morphology and altered expression profile of neurons may be very early phenotypes of RTT mouse models. Furthermore, subtle embryonic defects will be functionally amplified later in postnatal life, when overt RTT phenotypes will be established.

## Methods

### Animals

Female *Mecp2* heterozygous females [54] were purchased from Jackson Laboratories (strain name: B6.129P2(C) − Mecp2^tm1.1Bird/J^, stock number 003890). *Mecp2* mutant hemizygous males (*Mecp2* -/Y, also indicated as *Mecp2* null) and wild type (WT) littermates used as controls were obtained by mating heterozygous females with WT males (C57BL6/J background) for at least 12 generations. The national or institutional guidelines were used for the care and use of animals, and approval for the experiments were obtained from the ethical committees of the Italian Ministry of Health and the UK Home Office.

Genomic DNA was extracted from tail tips of embryos and immediately genotyped by polymerase chain reaction to determine Mecp2 deletion (according to [54]) and mice gender. The gender was determined using primers mSRY.F 5′-CCCAGCAGAATCCCAGCATGC-3′ and mSRYl.R 5′-TCCTGTCCCACTGCAGAAGGT-3′ specific for the Y chromosome-linked *Sry* gene.

Cortices from each embryo were separately dissected from those of the other littermates.

### Neuronal primary cultures

Dissociated cells were prepared by dissecting out cortices from individual embryos at embryonic day 15 (E15). The day of insemination (i.e. the appearance of a vaginal plug) is designated as embryonic day 0 (E0).

Cortices were dissected under a stereoscope in sterile conditions and placed in phosphate buffered saline (PBS) without calcium and magnesium, supplemented with 33 mM glucose. Cells were then cultured as previously described [61]. Briefly, the dissected areas were enzymatically dissociated by incubation for 20 min (min) at 37 °C in a papain solution (Warthington, 20 U/ml, Milan, Italy) in Earle's balance salts containing 1 mM EDTA (Sigma-Aldrich, Milan, Italy), 1 mM cysteine (Sigma-Aldrich) and 0,01 % pancreatic DNAse (Sigma-Aldrich). After addition of 1 mg/ml of bovine serum albumin (Sigma-Aldrich) and 1 mg/ml ovomucoid (Sigma/Aldrich) the cells suspensions were centrifuged 5 min at 800 rpm, resuspended in plating medium and counted [62]. For the viable cell count, cell suspension was diluted 1:1 with 0,1 % trypan blue dye (Sigma Aldrich) and loaded into a disposable cell counting chamber-slide. Cell concentration was determined on the basis of the total cell count, the dilution factor and the trypan blue dye exclusion.

Dissociated cells were plated at a density of 1 × 10^5^/cm^2^ in 2 cm^2^ cell cultures dishes (Corning) coated with 15 μg/ml of poly-D-lysine dissolved in water (Sigma-Aldrich).

Cultures were grown in serum-free Neurobasal medium (Life technologies, Milan, Italy), supplemented with B27 (Life technologies), 2 mM L-glutamine (Sigma-Aldrich), penicillin (50U/ml, Sigma-Aldrich) and streptomycin (50 μg/ml, Sigma-Aldrich). Cells were maintained for 3 days in vitro (DIV) at 37 °C in a humidified incubator in presence of 5 % CO_2_, before experimental manipulation. For each experimental point, cultures were prepared at least in independent triplicates, and were repeated using distinct culturing sessions.

Only primary cultures deriving from male embryos (see above) were then selected for subsequent analyses.

### Immunocytochemistry and analysis of morphometric parameters

For immunocytochemical analyses cultured cells were fixed in 4 % paraformaldehyde in PBS, for 30 min at room temperature (RT), washed three times in PBS, and then permeabilized for 20 min in PBS containing 0,1 % Triton-X-100 and 10 % normal goat serum (NGS). Cells were treated with blocking solution [10 % NGS, 0,1 % bovine serum albumine (BSA) in PBS] at RT for 1 h and incubated with the primary antibody in antibody solution (PBS containing 0,1 % BSA) overnight at 4 °C. The following antibodies were used at the indicated dilutions: monoclonal antibody against neuron specific class III β-tubulin (Tuj1, Covance, Milan, Italy) 1:500, polyclonal rabbit antibody anti-γ-Aminobutyric acid (GABA, Sigma, 1:500) and polyclonal rabbit antibody against Glial Fibrillary Acidic Protein (GFAP, Dako, Z 0334) 1:450.

Cells were washed 3 times in PBS, and then incubated for 2 h at RT with fluorescent-labeled secondary antibodies (Alexa Fluor Goat anti-rabbit, and Alexa Fluor Goat anti-mouse, Life Technologies) diluted 1:400 in antibody solution.

Cells were then counterstained with DAPI (nuclear stain, 1:1000) for 10 min, washed with PBS and mounted with oil mounting solution (Mowiol). As negative controls, some cells were processed as described above, but without primary antibody.

For morphological analyses, cells fixed and permeabilized as above, were incubated with the monoclonal antibody against neuron specific class III β-tubulin (anti-Tuj1, 1:750, Covance) and with the fluorescent-labeled secondary antibody, both diluted in PBS containing 10 % NGS, for 2 h at RT. After Tuj1 staining, cell-culture slides were analyzed with a Leica microscope (Leica DM6000B) using the software Leica Application Suite (LAS AF). Images were obtained by using a 20x objective and captured with a HAMAMATSU Digital Camera C10600 ORCA R2. Measurements of neurites length have been performed by the image-processing software Image J. Images were pre-processed to optimize illumination and contrast. The length of the neurites was estimated by measuring the length of a line manually drawn from the soma to the end of the primary neurites (neurites that originate directly from soma) using the “Measure” function of the software.

The primary branching was measured by the Image J plugin “Sholl analysis”. Its internal algorithm creates a series of concentric circles around the soma of the neuron and counts the number of neurites crossing these circles. Only clearly visible cells were analysed to prevent inaccurate scoring. For morphological analyses, a total of 15 fields for each cell-culture condition were used from at least three independent culture wells.

A total of 300 neurites/well were traced from Tuj1 positive neurons to measure their length. The analyses were carried out “blind” to avoid any subjective influences during measurements.

### RNA isolation, sequencing and RT-PCR

Total RNA was prepared from neuronal primary cultures using TRIZOL Reagent (Invitrogen) with addition of 150 μg of RNAase-free glycogen in order to maximize yield. RNA was then treated with RNase-free DNase (Ambion, DNA-free kit).

To minimize inter-individual variability, equal amount of quality checked RNAs from three individual embryos were mixed to constitute a pooled sample. With this procedure we obtained 3 samples/genotype with 1:1 proportion between RNAs derived from each Mecp2 null embryo (males) and those isolated from WT littermates. Samples were labeled as WT1-3 or N1-3.

RNA libraries were constructed using the TruSeq Stranded mRNA Sample preparation kit (Illumina), and single-strand sequenced on Illumina Hiseq2500 platform at the Istituto di Genomica Applicata (Udine, Italy). The first purification step isolates the poly-A containing mRNA molecules using poly-T oligo attached magnetic beads. During the second elution the poly-A RNA is also fragmented and primed for cDNA synthesis.

To verify the changes in expression of the intergenic region between *Mecp2* and *Irak1* loci, RT-PCR was performed on independent samples from additional animals. For each sample 1 μg of total RNA prepared from neuronal primary cultures was digested with DNase (Ambion, DNA-free kit) and reverse-transcribed by random-priming using SuperScript II first strand kit (Invitrogen). The following mouse primers were used for PCR analysis: inter5F 5′-GTCCCTAATGGGAGAACCGA-3′ and inter2R 5′-AGGTGAGTGGCAATGGCTAA-3′; inter3F 5′-CCCAGCTCCTCTCTTGAACAC-3′ and inter3R 5′-CTTTCAGCTGGCCAAACAAGG-3′. As loading control, GAPDH primers have been used: gapdhup 5′- TGCACCACCAACTGCTTAGC-3′ and gapdhlw 5′-TCTTCTGGGTGGCAGTGATG-3′.

### RNA-sequencing data analysis

Raw reads were processed with Trimmomatic [63] in order to remove low quality nucleotides and adapters. The minimum phred quality score for bases was set to 35 and only reads with a minimum length of 25 bp were retained after trimming. The high quality reads were aligned against the Mus musculus reference genome sequence (GRCm38) with TopHat [64] version 2.0.9. The resulting alignment files were used as input for HTSeq-count [65] version 0.5.4p2 together with the GRCm38 annotation file to calculate gene expression values (read counts). Genes with very low read counts or those that were too variable among the replicates were removed with HTSFilter [66]. Differential expression analysis was performed in R with the package TCC [67] in combination with edgeR [68]. The coordinates of the intergenic region between *Irak1* (ENSMUSG00000031392) and *Mecp2* (ENSMUSG00000031393) and the number of the mapped reads in each sample were obtained with bedtools (v2.17.0).

Expression profiles of the differentially expressed genes were used to perform a K-mean clustering with MeV [69] and 7 clusters were identified by using Pearson Correlation as distance metric.

Gene Ontology (GO) and functional annotation enrichment analysis was carried out using Database for Annotation, Visualization and Integrated Discovery (DAVID) bioinformatics resources 6.7 (http://david.abcc.ncifcrf.gov/) maintained by National Institute of Allergy and Infectious diseases (NIAID), NIH. We used both DAVID web interface as well as custom made Python script implementing DAVID-web services in our in-house built Transcriptator software [70].

Transcriptator helps in determining automatic and reproducible GO and functional annotation results for the differentially expressed list of genes derived from transcriptomic analysis of data. Currently, DAVID provides annotation for 40 different categories. In our computational pipeline we includes GO terms, protein-protein interactions, protein functional domains, bio-pathways, sequence general features and gene functional summaries. Using DAVID web application, we exploited functional annotation enrichment as well as functional annotation clustering tools to obtain clusters of differentially expressed genes based on common functionalities. For the GO and functional annotation enrichment, we defined a stringent Ease score (*p*- value) ≤0.05 and Count ≥5, which is basically a modified Fischer exact test *p*-value to examine more conservatively the enrichment situation.

For neuronal specific genes, we carried out functional annotation clustering based on the algorithm in DAVID, which hypothesizes that similar annotations should have similar gene members. It integrates the Kappa statistics to measure the degree of common genes between two annotations and fuzzy heuristic clustering to classify groups of similar annotations according to kappa values. In easier terms, the higher the number of shared annotations terms, the greater the probability that genes will be grouped together [71, 72].

For the functional annotation clustering implemented algorithm and utilized in DAVID web application we selected a stringent cut-off Ease score ≤0.05 and an enrichment score value ≥ 1. We also carried out GO annotation analysis through AmiGO 2 (version: 2.2.0 amigo2b) with GO database release 2015-06-06. Jvenn software was used to create Venn diagrams [73].

## Availability of supporting data

The datasets supporting the results of this article are included within the article as Additional file 5.

## Additional files

Additional file 1:
**Immunocytochemical characterization of cultured cortical cells with neuronal and glial markers.** Representative images of cortical cells dissociated from E15 WT embryos, fixed after 3 DIV and analysed using DAPI to stain nucleus (blue), anti-Tuj1 antibody to stain neurons (red), anti- GABA antibody to stain GABAergic neurons, anti-GFAP to stain structural glial/astrocytic protein (green). Merged pictures show relative proportions and localizations of neuronal and glial cells within a particular microscopic field. Magnification: 20x; Leica DMI6000 B inverted microscope. (PDF 3158 kb) Additional file 2:
**Heatmap of the expression profiles of 50 random genes from WT and null samples.** Green to red gradient has been used to represent low and high expressed genes, respectively (FPKM values). On the top, the dendrogram represents the result of the hierarchical clusterization of the samples by using the euclidean distance obtained with the expression profile of all the genes in the genome. (PDF 408 kb) Additional file 3:
**Expression analysis of the genomic region from** ***Mecp2*** **-3′UTR to** ***Irak1-*** **5′UTR in WT and** ***Mecp2*** **null samples.** Left panel: From the left to the right of the figure the following elements are shown: the coverage (grey bars) and mapped reads (red bars) for WT3, WT2, N3 and N2 samples, the schematic structure of the 3′-end of *Mecp2* and 5′-end of *Irak1* (with UTRs marked as thin rectangles, exons as thick rectangles and introns as lines). The grey lines connecting reads represent spliced reads. The horizontal dotted box highlights the intergenic region between *Irak1* and *Mecp2* to show the lack of mapped reads in the WT samples and the presence of spliced reads connecting the two loci in the null mutants. Right panel: RT-PCR using two set of primers spanning the intergenic region was performed on independent WT (WT4 and 5) and null samples (N4, 5 and 6). GAPDH transcript was used as internal control. (PDF 129 kb) Additional file 4:
**Comparison of DE genes defined as unmapped with GeneChip microarray used in Cahoy’s study.** Venn diagram comparing Affymetrix Mouse Genome 430 2.0 Array GPL1261 probe set (GSE9566) with unmapped genes identified in this study. Common genes are in total 166. (PDF 26 kb) Additional file 5:
**Lists of the 490 differentially expressed genes distributed across neural cell types.** DE genes found in this study have been assigned to specific cell types accordingly to [31]. Specific cellular markers for each cell type are shown in the related sheet at the top of included tables. WT are wild type samples, N are *Mecp2* null samples. For unmapped genes we arbitrarily consider the microglia gene CD11B as a marker. °*Irak1* has been found always up-regulated in *Mecp2* null mouse brains (Bird strain). In the table of neuronal genes results of comparison with dataset of 4125 developmentally regulated (E7 vs P30) genes in WT mouse cortex [37] are shown in the last column. In particular, each shared gene is shown with the developmental stage of enrichment reported by Dillman and co-workers. In the table of neuronal genes, results of comparison with 3 datasets of genes whose expression is altered in specific Mecp2 null brain regions are provided. The hypothalamus dataset [30] is made of genes whose expression has been found altered both in transgenic and null mice (2582 genes, red) together with those altered only in null mice (369 genes, blue). The concordance/discordance labeling is referred to deregulation in null mice. In the astrocyte table results of comparison with dataset of [29] are shown in the last column. *Fxyd1 is an already known Mecp2-target gene found up-regulated also in [55]. (XLSX 47 kb) Additional file 6:
**(A and B):Functional annotation clustering of oligodendrocytes specific differentially expressed genes.** A) GO-based analysis shows that most significantly enriched biological processes (*p*-value ≤ 0.05) of DE genes mapped to oligodendrocytes are specifically related to this cell-type. B) Panther family-based analysis shows that most significantly enriched terms (*p*-value ≤ 0.05) include signaling molecules (tetraspanin- PTHR19282) and basic helix-loop-helix transcription factor proteins (basic helix-loop-helix protein neurogenin-related-PTHR19290) having a documented role in oligodendrocytes differentiation. (PDF 252 kb) Additional file 7:
**Functional annotation clustering of differentially expressed unmapped genes.** GO-based analysis shows that most significantly enriched biological process (*p*-value ≤ 0.05) of DE unmapped genes is an immunomodulator-mediated signaling pathway related to broadly ranging cellular activities. (PDF 143 kb) Additional file 8:
**Functional annotation analysis of neuron enriched differentially expressed genes through DAVID and Amigo2.** GO and functional annotation enrichment analysis using DAVID (first sheet), for the given list of neuronal specific genes. It provides enriched functional terms with *P*-value and adjusted *p*-value (through multiple test correction). It separately provides enriched functional annotation terms (significant *p*-values) in the categories of GO terms, UP-SEQ-Features, SP-PIR-keywords, SMART, PIR, Interpro domains and PANTHER annotations. The multiple test correction for these terms does not provide significant *p*-values due to low number of genes in each category. GO annotation analysis through AmiGO 2 (second sheet). Significant GO terms for biological process, molecular functions and cellular locations are retrieved for neuronal specific genes in our differential expressed gene list using Bonferroni multiple test correction method, with a *p*-value cutoff < 0.05. (XLSX 69 kb)

## Abbreviations

CNS: central nervous system
DE: differentially expressed
E15: embryonic day 15
Mecp2: methyl CpG binding protein 2
RTT: Rett Syndrome
