# Supplementary figures and images for: Effects of Mecp2 loss of function in embryonic cortical neurons: a bioinformatics strategy to sort out non-neuronal cells variability from transcriptome profiling
Source: BMC Bioinformatics. 2016 Jan 20;17(Suppl 2):14. doi: 10.1186/s12859-015-0859-7 (PMC4959389; doi:10.1186/s12859-015-0859-7)

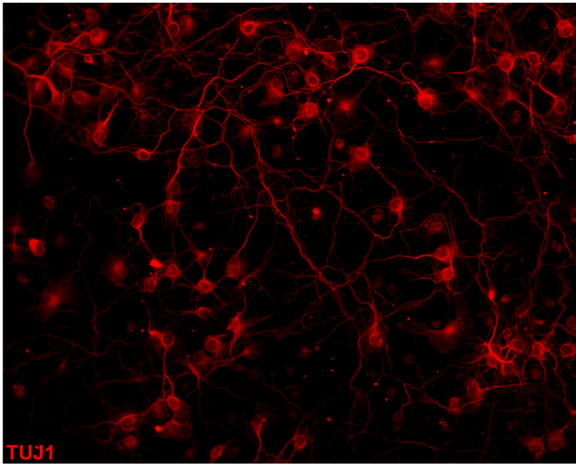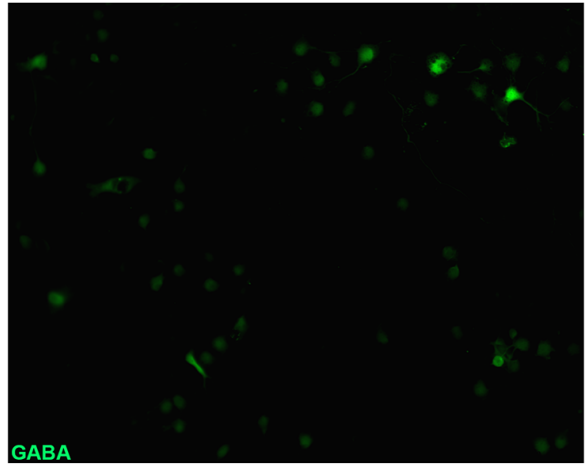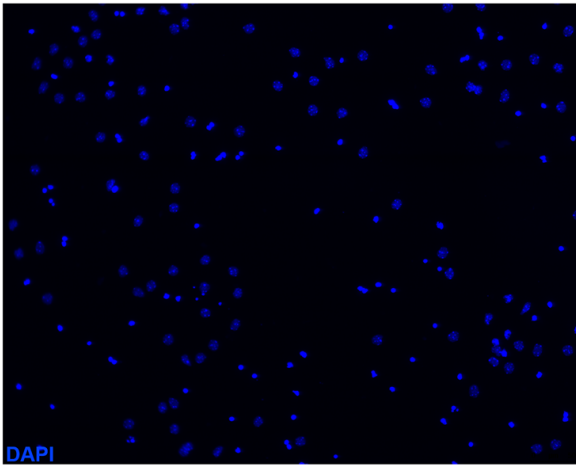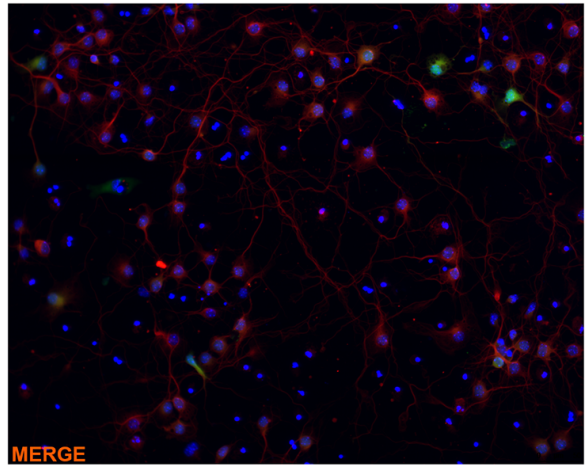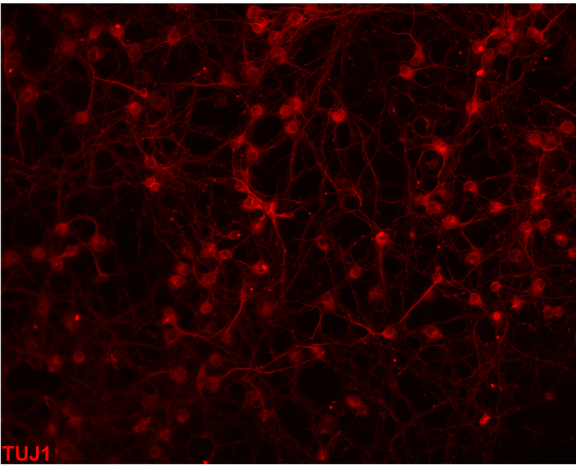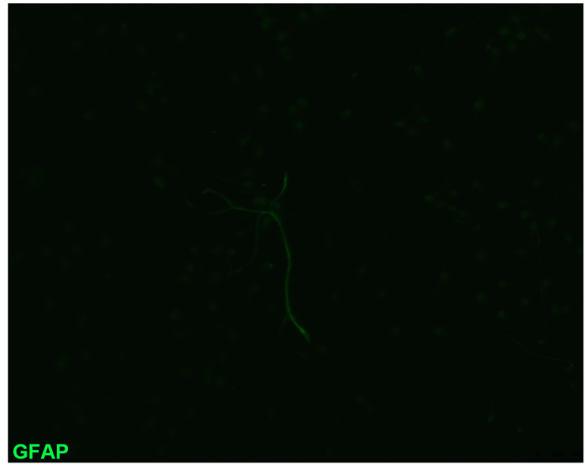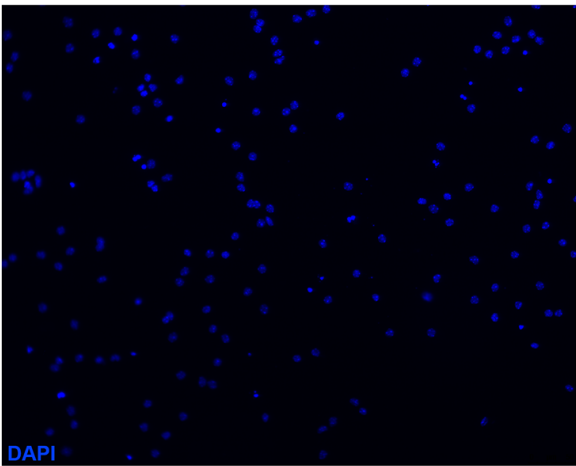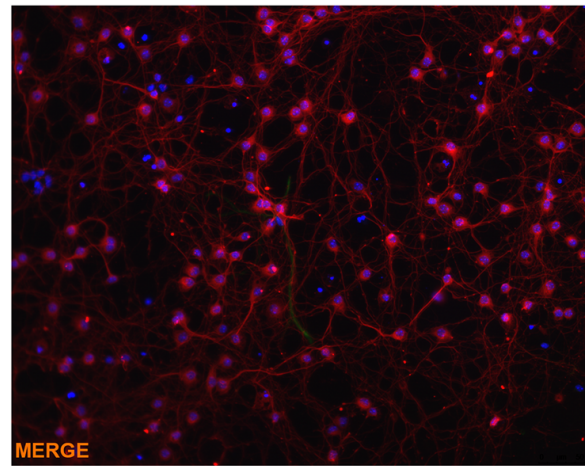

Supplement: Additional file 1: — Immunocytochemical characterization of cultured cortical cells with neuronal and glial markers. Representative images of cortical cells dissociated from E15 WT embryos, fixed after 3 DIV and analysed using DAPI to stain nucleus (blue), anti-Tuj1 antibody to stain neurons (red), anti- GABA antibody to stain GABAergic neurons, anti-GFAP to stain structural glial/astrocytic protein (green). Merged pictures show relative proportions and localizations of neuronal and glial cells within a particular microscopic field. Magnification: 20x; Leica DMI6000 B inverted microscope. (PDF 3158 kb) [file 12859_2015_859_MOESM1_ESM.pdf]

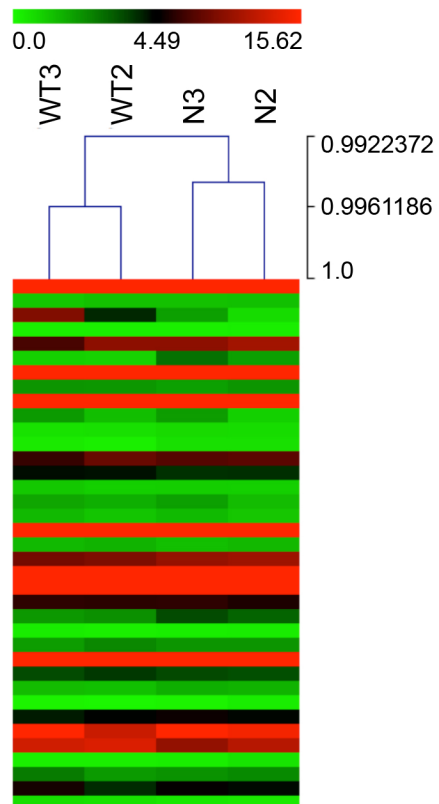

Supplement: Additional file 2: — Heatmap of the expression profiles of 50 random genes from WT and null samples. Green to red gradient has been used to represent low and high expressed genes, respectively (FPKM values). On the top, the dendrogram represents the result of the hierarchical clusterization of the samples by using the euclidean distance obtained with the expression profile of all the genes in the genome. (PDF 408 kb) [file 12859_2015_859_MOESM2_ESM.pdf]

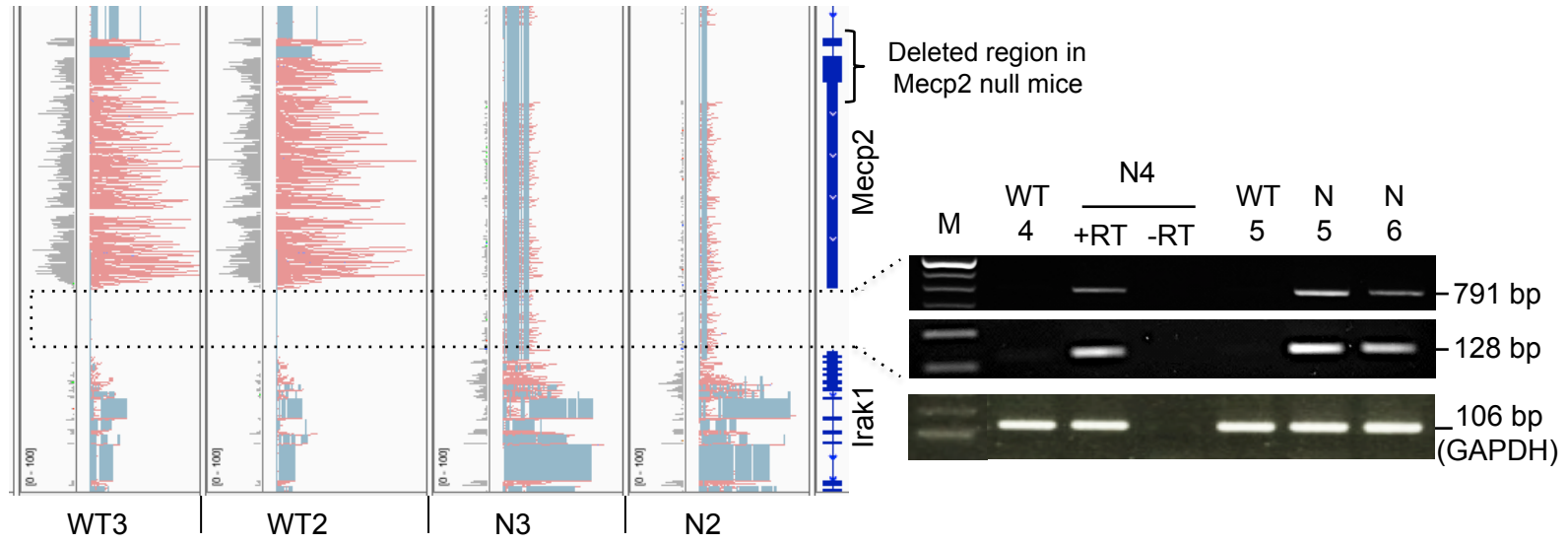

Supplement: Additional file 3: — Expression analysis of the genomic region from Mecp2 -3′UTR to Irak1- 5′UTR in WT and Mecp2 null samples. Left panel: From the left to the right of the figure the following elements are shown: the coverage (grey bars) and mapped reads (red bars) for WT3, WT2, N3 and N2 samples, the schematic structure of the 3′-end of Mecp2 and 5′-end of Irak1 (with UTRs marked as thin rectangles, exons as thick rectangles and introns as lines). The grey lines connecting reads represent spliced reads. The horizontal dotted box highlights the intergenic region between Irak1 and Mecp2 to show the lack of mapped reads in the WT samples and the presence of spliced reads connecting the two loci in the null mutants. Right panel: RT-PCR using two set of primers spanning the intergenic region was performed on independent WT (WT4 and 5) and null samples (N4, 5 and 6). GAPDH transcript was used as internal control. (PDF 129 kb) [file 12859_2015_859_MOESM3_ESM.pdf]

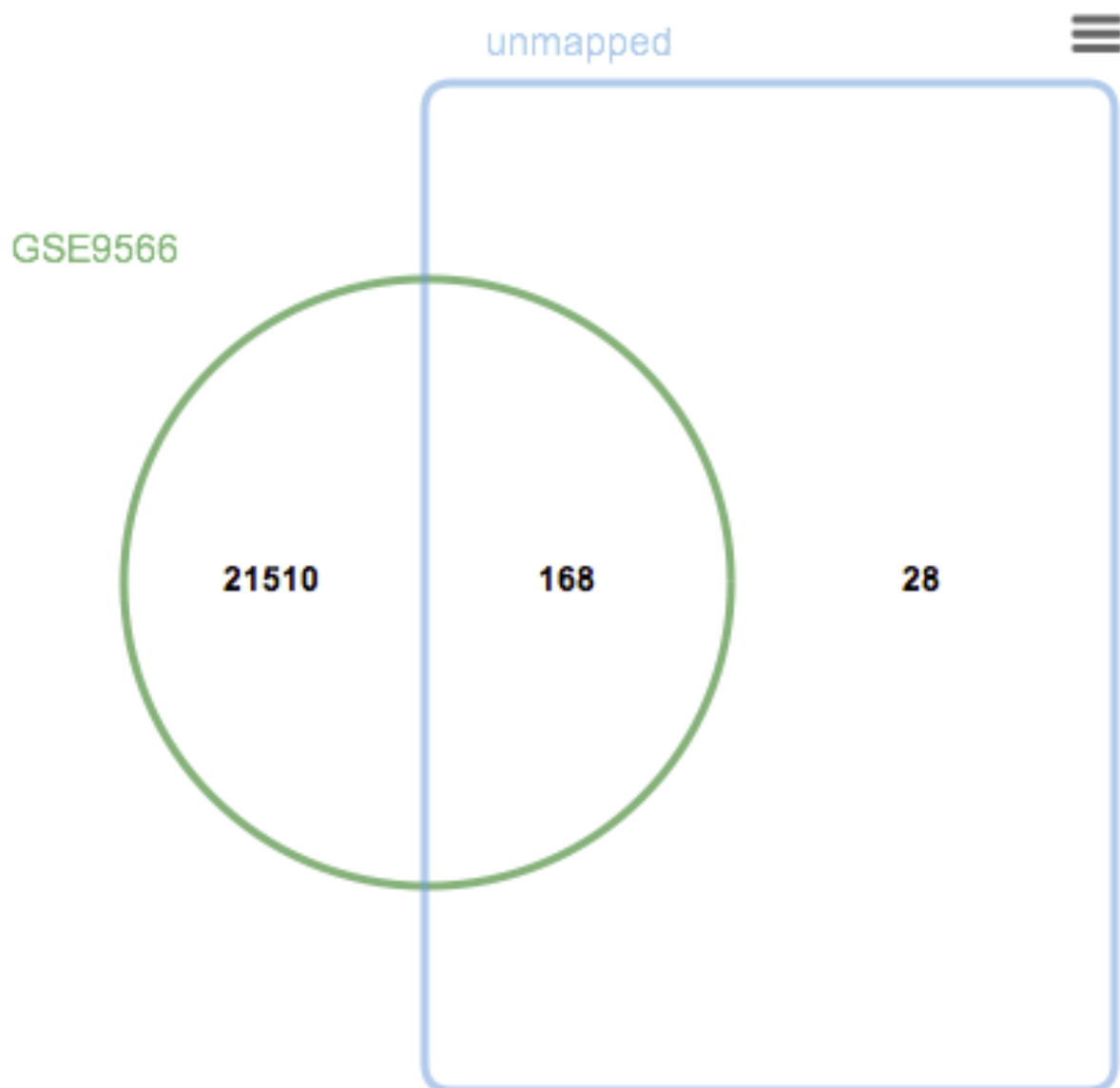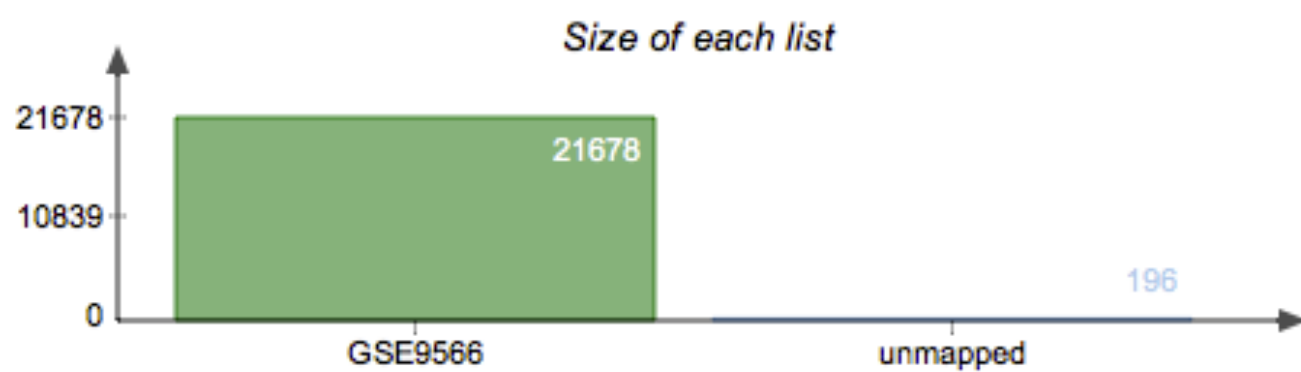

Supplement: Additional file 4: — Comparison of DE genes defined as unmapped with GeneChip microarray used in Cahoy’s study. Venn diagram comparing Affymetrix Mouse Genome 430 2.0 Array GPL1261 probe set (GSE9566) with unmapped genes identified in this study. Common genes are in total 166. (PDF 26 kb) [file 12859_2015_859_MOESM4_ESM.pdf]

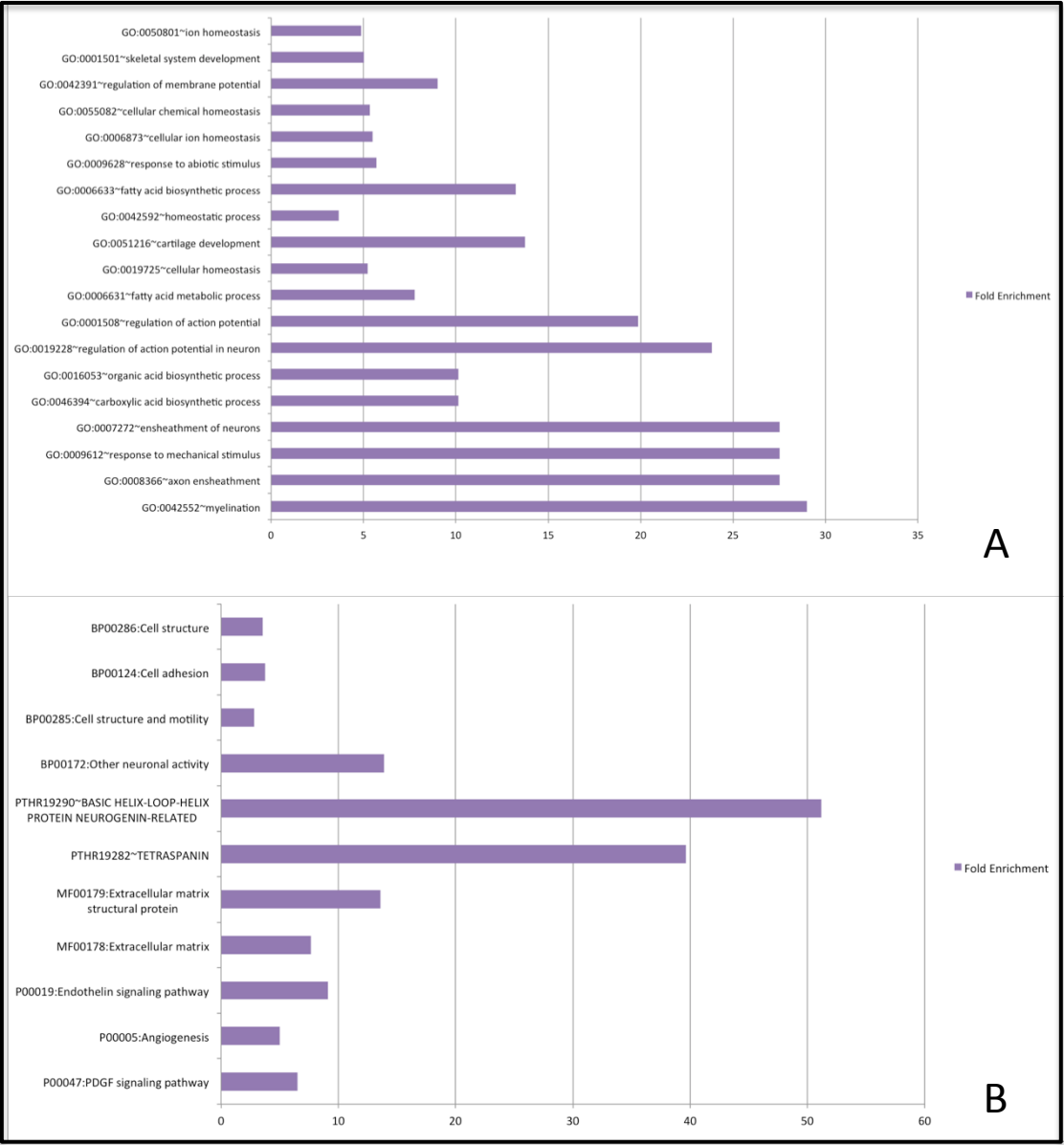

Supplement: Additional file 6: — (A and B):Functional annotation clustering of oligodendrocytes specific differentially expressed genes. A) GO-based analysis shows that most significantly enriched biological processes (p-value ≤ 0.05) of DE genes mapped to oligodendrocytes are specifically related to this cell-type. B) Panther family-based analysis shows that most significantly enriched terms (p-value ≤ 0.05) include signaling molecules (tetraspanin- PTHR19282) and basic helix-loop-helix transcription factor proteins (basic helix-loop-helix protein neurogenin-related-PTHR19290) having a documented role in oligodendrocytes differentiation. (PDF 252 kb) [file 12859_2015_859_MOESM6_ESM.pdf]

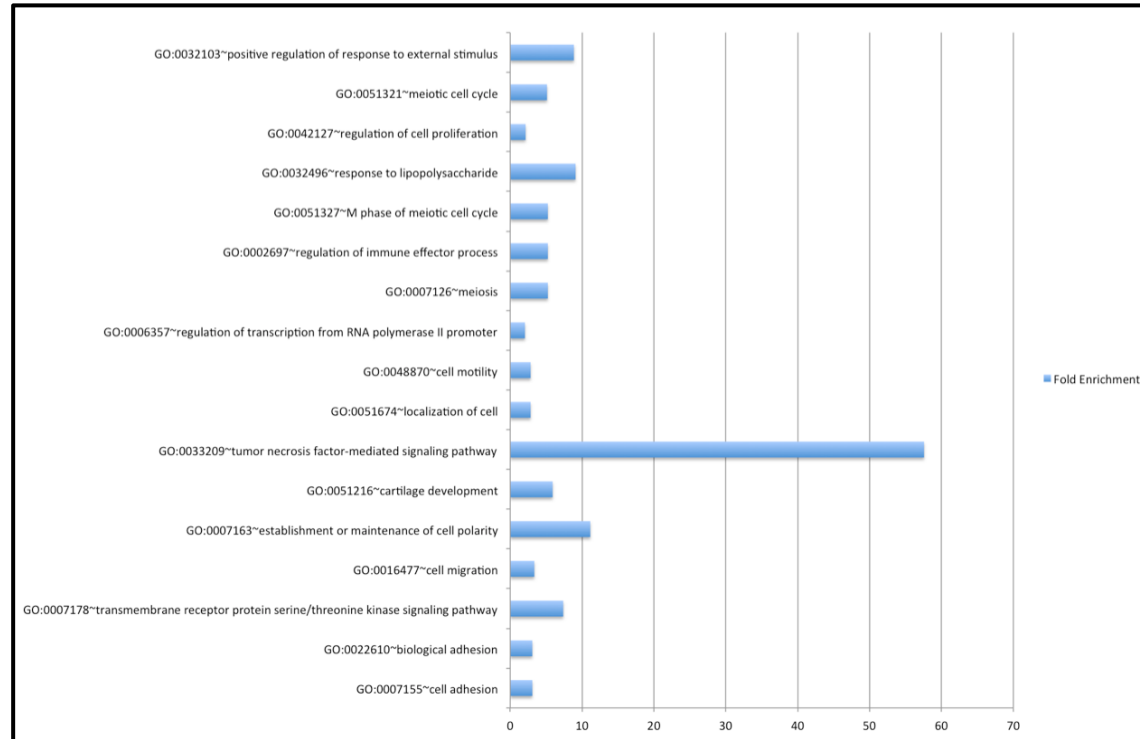

Supplement: Additional file 7: — Functional annotation clustering of differentially expressed unmapped genes. GO-based analysis shows that most significantly enriched biological process (p-value ≤ 0.05) of DE unmapped genes is an immunomodulator-mediated signaling pathway related to broadly ranging cellular activities. (PDF 143 kb) [file 12859_2015_859_MOESM7_ESM.pdf]
